# Magnetic-driven hydrogel microrobots for promoting osteosarcoma chemo-therapy with synthetic lethality strategy

**DOI:** 10.3389/fchem.2024.1386076

**Published:** 2024-04-04

**Authors:** Yining Tao, Leike Li, Xiyu Yang, Shiyu Yin, Zhanxiang Zhang, Haoyu Wang, Ruochen Pu, Zongyi Wang, Qi Zhang, Haoran Mu, Chenqiong Wu, Jin He, Liu Yang

**Affiliations:** ^1^ Department of Orthopedics, Shanghai General Hospital, School of Medicine, Shanghai Jiao Tong University, Shanghai, China; ^2^ Shanghai Bone Tumor Institution, Shanghai, China; ^3^ State Key Laboratory of Robotics and System, Harbin Institute of Technology, Harbin, China; ^4^ Community Health Service Center, Shanghai, China; ^5^ Jintan Hospital Affiliated to Jiangsu University, Changzhou, Jiangsu Province, China; ^6^ Department of Biochemistry and Molecular Cell Biology, Shanghai Key Laboratory for Tumor Microenvironment and Inflammation, Shanghai Jiao Tong University School of Medicine, Shanghai, China

**Keywords:** microrobots, magnetic-driven, drug delivery system, MYC, synthetic lethality, osteosarcoma, chemotherapy

## Abstract

The advancements in the field of micro-robots for drug delivery systems have garnered considerable attention. In contrast to traditional drug delivery systems, which are dependent on blood circulation to reach their target, these engineered micro/nano robots possess the unique ability to navigate autonomously, thereby enabling the delivery of drugs to otherwise inaccessible regions. Precise drug delivery systems can improve the effectiveness and safety of synthetic lethality strategies, which are used for targeted therapy of solid tumors. MYC-overexpressing tumors show sensitivity to CDK1 inhibition. This study delves into the potential of Ro-3306 loaded magnetic-driven hydrogel micro-robots in the treatment of MYC-dependent osteosarcoma. Ro-3306, a specific inhibitor of CDK1, has been demonstrated to suppress tumor growth across various types of cancer. We have designed and fabricated this micro-robot, capable of delivering Ro-3306 precisely to tumor cells under the influence of a magnetic field, and evaluated its chemosensitizing effects, thereby augmenting the therapeutic efficacy and introducing a novel possibility for osteosarcoma treatment. The clinical translation of this method necessitates further investigation and validation. In summary, the Ro-3306-loaded magnetic-driven hydrogel micro-robots present a novel strategy for enhancing the chemosensitivity of MYC-dependent osteosarcoma, paving the way for new possibilities in future clinical applications.

## 1 Introduction

Osteosarcoma is a highly malignant tumor that originates from bone tissue and has a high propensity for metastasis (Ritter and Bielack, 2010). Although the underlying mechanisms of osteosarcoma are still poorly understood, we know that its clinical outcome is usually unfavorable, and enhancing the survival rate of patients remains a major challenge. Currently, the treatment options for osteosarcoma mainly consist of limb-sparing surgery, neoadjuvant chemotherapy, personalized therapy, and tumor segment bone devitalization and replantation. These treatment modalities can not only preserve the patient’s limbs, but also increase the patient’s long-term survival rate (over 5 years) to 80% (Chen et al., 2021). However, in recent years, the treatment of osteosarcoma has reached a plateau, especially for chemotherapy-resistant patients, who require new drugs and treatment strategies (Gill and Gorlick, 2021; Meltzer and Helman, 2021).

When exploring new treatment approaches, we need to consider the possible toxicity and side effects of drugs on normal tissue cells. Efficient drug delivery systems are regarded as one of the effective means to improve the efficacy and safety of tumor treatment. Drug delivery system (DDS) is a technical system that can control the distribution of drugs in the body in a spatiotemporal and dosing manner. DDS can improve the targeting and selectivity of drugs, enhance the stability and bioavailability of drugs, achieve synergistic enhancement and multidimensional treatment of drugs, thereby improving the effect and safety of tumor treatment (Vargason et al., 2021; Yi et al., 2022; Li et al., 2023).

Micro/nano-robot drug delivery system is an emerging drug delivery technology that employs artificial robots at micro/nano scale as drug carriers, which are propelled by external energy fields or self-motility, to navigate and control precisely in the body, and deliver drugs to specific lesion sites (Soto et al., 2020). Micro/nano-robots can utilize different driving modes (such as chemical, electromagnetic, ultrasound, photothermal, etc.) to achieve autonomous movement and navigation in complex biological environments such as blood vessels, lymph, and tissues, and enhance the targeting and selectivity of drugs (Choi et al., 2021). In addition, micro/nano-robots can also load multiple drugs or biologics simultaneously, and regulate the release order and ratio of drugs, to achieve synergistic effects between drugs, and overcome drug resistance and side effects. Among the various types of micro/nano-robots, magnetic-driven hydrogel micro-robots are a kind of capsule-type micro-robots based on hydrogels, which can use external magnetic fields to achieve precise movement control and navigation, and overcome the fluid dynamic resistance and Brownian motion interference in low Reynolds number fluids (Chen et al., 2021). Moreover, magnetic-driven hydrogel micro-robots exploit the high water absorption and porosity of hydrogels to achieve efficient drug loading and responsive drug release, and avoid the premature leakage and non-specific effects of drugs. Furthermore, due to the high biocompatibility and biodegradability of hydrogels, this drug delivery system carrier reduces the toxicity and side effects on tissue cells, and has high safety and bioavailability (Hu et al., 2020; Zhang et al., 2021).

Based on the advantages of magnetic-driven hydrogel micro-robots in drug delivery systems, it is of great significance to explore their application value in the diagnosis and treatment of osteosarcoma. We have used multi-omics methods to establish a molecular precision subtyping of osteosarcoma based on multi-omics data. Among them, MYC-driven osteosarcoma is characterized by genomic amplification and high expression of the MYC gene. This subtype of osteosarcoma has the worst prognosis, with a 5-year survival rate of less than 40%. Improving the prognosis of this subtype of patients or overcoming the overall prognosis “bottleneck” of osteosarcoma is the key link (Jiang et al., 2022). In tumors with MYC overexpression, tumor cells become dependent on MYC (Kalkat et al., 2018; Huang et al., 2021). At the same time, due to the difficult-to-target biological characteristics of MYC, targeting the downstream targets of MYC to form a synthetic lethal strategy may be more effective in this subtype of cancer patients (Nijman and Friend, 2013; Solvie et al., 2022; Sun et al., 2022). Tumor cells with MYC overexpression exhibit cell cycle dysregulation, which may make their proliferation particularly sensitive to the inhibition of an important regulator of cell cycle progression - cyclin-dependent kinase (CDK) (Icard et al., 2019; Baluapuri et al., 2020). Among them, inhibiting CDK1 can induce MYC-dependent apoptosis. However, targeting MYC’s synthetic lethality is different from other genes’ synthetic lethality. MYC, as an important transcription factor for cell cycle regulation, its synthetic lethal gene CDK1 plays an important role in every stage of the cell cycle and is a key factor for maintaining normal cell proliferation. Excessive inhibition of CDK1 may produce unpredictable toxic and side effects (Goga et al., 2007; Massacci et al., 2023; Wang et al., 2023). Using magnetic-driven non-targeted robots can theoretically alleviate the strong toxicity to the surrounding normal tissues and effectively increase the drug concentration in osteosarcoma tissues. In addition, some studies have shown that targeting CDK1 can reduce the efflux or metabolism of chemotherapy drugs by tumor cells, thereby increasing the accumulation and effect of chemotherapy drugs at the tumor site (Zeng et al., 2023). Therefore, combining magnetic-driven hydrogel micro-robots with CDK1 inhibition may be an effective method to improve the chemotherapy sensitivity of MYC-dependent osteosarcoma, and is expected to improve the prognosis of MYC-driven osteosarcoma patients.

To explore the value of combining magnetic-driven hydrogel micro-robots with CDK1 targeting in the treatment of osteosarcoma, we chose Ro-3306 (a CDK1 inhibitor) as the research drug. Ro-3306 has low water solubility and bioavailability in the body, which hinders its clinical application (Huang et al., 2023). However, by loading it on magnetic-driven hydrogel micro-robots, we can enhance its bioavailability. Our study will investigate the application value and research progress of magnetic-driven hydrogel micro-robots loaded with Ro-3306 in improving the chemotherapy sensitivity of MYC-dependent osteosarcoma, with the aim of improving the prognosis of MYC-driven osteosarcoma patients.

## 2 Materials and methods

### 2.1 Cell lines, reagents and cell culture

The human osteosarcoma cell lines SJSA, SAOS2, 143B, U2OS and HOS were cultured in high glucose Dulbecco’s Modified Eagle Medium (DMEM; Gibco, United States) supplemented with 10% fetal bovine serum (FBS; Wisent, Canada) and 1% Penicillin-Streptomycin-Glutamine (Wisent Inc., Cañada) in a humidified atmosphere of 5% CO2 and 95% air at 37°C. Cells were dissociated by TrypLETM Express (Gibco, Thermo Fisher Scientific Inc., United States) and passaged when they are 90%–100% confluent. Cell line authentication was performed on cells that used for *in vitro* and *in vivo* studies using Short Tandem Repeat (STR) DNA profiling and all cell lines were preserved at Shanghai Bone Tumor Institute (Shanghai, China). Reagents used in the study included: Ro-3306 (#HY-12529, MCE/MedChemExpress, United States), Doxorubicin hydrochloride (#HY-15142, MCE/MedChemExpress, United States). The antibodies used in this study were purchased as follows: anti-MYC (#ab78318, 1:1000), anti-GAPDH (#ab263962, 1:1000). HRP-conjugated goat anti-rabbit IgG (#L3042, 1:5000) and goat anti-mouse IgG (#101, 1:5000) secondary antibodies for Western blot were purchased from Signalway Antibody.

### 2.2 Stable gene overexpression

Constitutive overexpression of GFP in 143B cell line was achieved using lentiviral infection of pGMLV vector (modified from Genomeditech, dual Promoter EF1-ZsGreen1-T2A-Puro), selected with puromycin (2ug/mL). Lentivirus production was obtained from PEI transfection reagents (#26406, Polyseciences, United States) of HEK-293T cells with co-transfection of the packaging vectors pspax2 and pMD2. G along with the gene delivery vector. Viral supernatants were collected 72 h after transfection, underwent ultracentrifugation at 20,000 rpm for 25 h at 4°C to concentrate, and the virus pellets were resuspended in PBS. For infection, the viral pellets were added to cells in a dropwise manner in the presence of polybrene (10ug/mL). After 48h, medium containing the lentivirus was replaced and infected cells were selected by addition of puromycin (2ug/mL).

### 2.3 Colony formation

To assay cell growth, osteosarcoma cells were washed twice with PBS and plated onto 60 mm cell culture dishes at a density of 3000 cells per dish. Osteosarcoma cells were treated with or without drugs for 1 week, and the cell colonies were stained using crystal violet staining solution (Beyotime, China) according to the manufacturer’s instructions.

### 2.4 Calcein/AM-PI

For the detection of cell viability, osteosarcoma cells were plated onto 24-well plates at a density of 5000 cells per well. Osteosarcoma cells were treated with or without drugs for 3 days. Osteosarcoma cells were stained with Calcein/AM and propidium iodide (PI) (Beyotime, China) according to the manufacturer’s instructions. Briefly, cells were washed with PBS and then incubated with 2 μM Calcein/AM and 4.5 μM PI in PBS at 37°C for 30 min. After incubation, cells were washed with PBS and immediately observed under a fluorescence microscope. Live cells were identified by green fluorescence (Calcein/AM), and dead cells were identified by red fluorescence (PI). Images were acquired using Leica TCS SP8 Laser Scanning Confocal Microscope (Leica, United States) and were processed by Leica Application Suite X (LAS X; Leica, United States).

### 2.5 CellTiter-Glo Luminescent Cell Viability Assay

3D-Cell viability was assessed using the CellTiter-Glo Luminescent Cell Viability Assay (Promega, United States) following the manufacturer’s instructions. Briefly, cells were seeded in a 96-well plate at a density of 2 × 104 cells/well and incubated for 24 h. After treatment with or without drugs for 5 days, 100 μL of CellTiter-Glo reagent was added to each well and mixed for 2 min on an orbital shaker to induce cell lysis. The plate was then incubated at room temperature for 10 min to stabilize the luminescent signal. Luminescence was detected using SpectraMax M3 Microplate Reader (Molecular Devices, United States).

### 2.6 Three-dimensional cell culture

The cell pellet was resuspended in three-dimensional cell culture medium, which consisted of the following components: DMEM/F12 (Thermo Fisher Scientific, United States), supplemented with 1% penicillin-streptomycin (Gibco, United States), 10% fetal bovine serum (FBS; Wisent, Canada), 0.5% GlutaMax (Gibco, United States), 0.5% N2 supplement (Thermo Fisher Scientific, United States), 0.5% MEM Non-Essential Amino Acids Solution (Thermo Fisher Scientific, United States), 0.5% B-27^®^ Serum-Free Supplement (Thermo Fisher Scientific, United States), 8 mg/mL EGF (PeproTech, United States), 10 ng/mL bFGF (MCE, MedChemExpress, United States), 10 ng/mL IGF-1 (MCE, MedChemExpress, United States), and 5 ng/mL TGF-beta 3 (MCE, MedChemExpress, United States). The prepared cell suspension was then added to an ultra-low attachment U-bottom 96-well plate (Corning, United States). The cell count in each well was adjusted to be between 300 and 5000 cells. The plate was then placed in a 37°C incubator with a 5% carbon dioxide atmosphere. Each well received 200 μL of the medium, which was changed every 24–36 h.

### 2.7 Western blotting

Proteins were extracted using radio immunoprecipitation assay (RIPA) lysis buffer (Beyotime, China) for total protein. Tissues were homogenized in RIPA lysis buffer (Beyotime, China) with protease and phosphatase inhibitors. Cells were washed with PBS and lysed in RIPA buffer. Lysates were centrifuged at 12,000 g for 30 min at 4°C, and the supernatant was collected. The protein concentration was measured using a Pierce BCA Protein Quantification Kit (#23325, Thermo Fisher Scientific, United States) and a SpectraMax M3 Microplate Reader (Molecular Devices, United States). Proteins were separated by sodium dodecyl sulfate polyacrylamide gel electrophoresis (SDS-PAGE) and transferred to 0.45 μm polyvinylidene fluoride (Millipore, United States) using a Mini-PROTEAN Tetra Vertical Electrophoresis Cell and a Mini Trans-Blot Module for tank transfer system with a PowerPac HV Power Supply (Bio-Rad, United States). The membrane was blocked with 5% nonfat dry milk in Tris-buffered saline solution with 0.1% Tween-20 (TBS-T) for 1 h at room temperature and then incubated with specific primary antibodies overnight at 4°C. Subsequently, the membranes were washed with TBS-T for 10 min three times, followed by incubation with HRP-conjugated secondary antibodies for 1 h at room temperature. Actin was used as the protein loading control. Protein signals were detected with SuperSignal West Femto Maximum Sensitivity Substrate (Thermo Fisher Scientific, United States) and imaged using a chemiluminescence imaging system Amersham Imager 600 (GE Healthcare, United States) and Tanon 5200 (Tanon, China).

### 2.8 Synthesis and characterization of magnetic-driven hydrogel micro-robot drug delivery system

**FIGURE 1:**
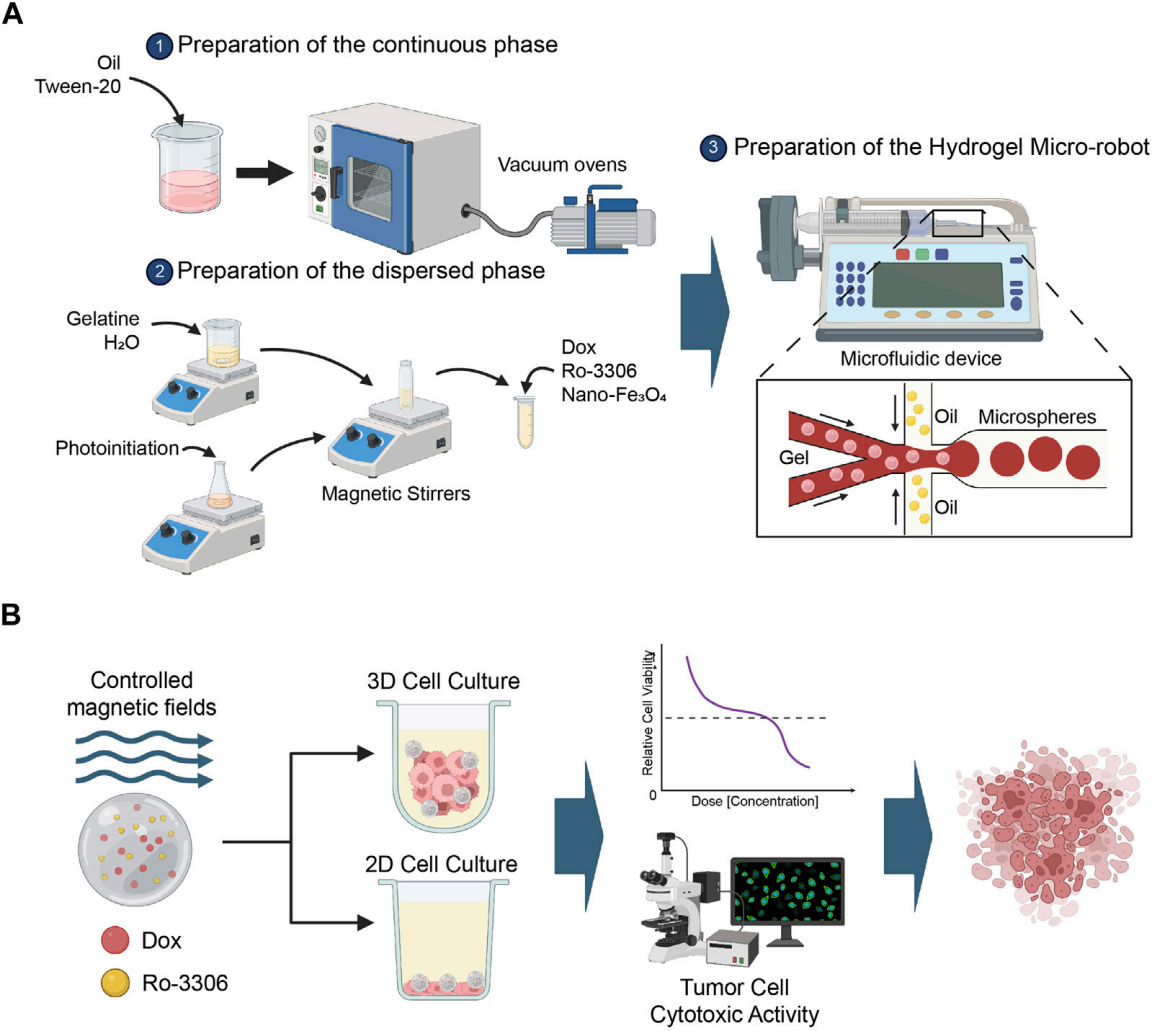
Methodology Scheme. **(A)** This study utilizes droplet microfluidic technology to construct gelatin microrobots. In the realm of droplet microfluidics, droplet formation is primarily governed by the interplay of tension differences between the continuous and dispersed phase fluids. The combined effect of surface tension and shear forces at the interface of these two phases gives rise to droplets. The accompanying figure presents a schematic representation of the fabrication of gelatin microspheres via a flow-focusing device. Created with BioRender.com. **(B)** This section provides an overview of the application of the magnetic-driven hydrogel micro-robots, developed in this study, for the treatment of osteosarcoma cell cultures under both two-dimensional and three-dimensional conditions. Post a 3-day culture period of the osteosarcoma cells, using either a conventional culture medium or a specially formulated medium for three-dimensional cell culture, the drug-loaded magnetic-driven hydrogel micro-robots are deployed for precision drug delivery, guided by the manipulation of magnetic field intensity. Upon conclusion of the experiment, a multifunctional enzyme marker is employed to assess cell viability, while a fluorescence microscope is utilized to monitor cell survival or apoptosis. Created with BioRender.com.

In this study, droplet microfluidic technology was employed to fabricate gelatin microrobots. In droplet microfluidics, the formation of droplets primarily depends on the tension difference between the continuous phase and the dispersed phase fluids. Droplets are formed at the interface of the two phases due to the combined action of surface tension and shear force. The schematic diagram of the preparation of gelatin microspheres through a flow focusing device is shown in Figure 1A. Specifically, vegetable oil was taken and Tween-20 was added to obtain the continuous phase fluid (1000:1). 333 mg of gelatin (gel strength: ∼250 g Bloom, Aladdin, China) was dissolved in 7 mL of pure water, and 20 mg of photoinitiator 2-hydroxy-4’- (2-hydroxyethoxy) -2-methylpropiophenone was dissolved in 3 mL of ddH2O. The solutions were mixed evenly to obtain 10 mL of gelatin aqueous solution with a photoinitiator. 0.9 mL of gelatin aqueous solution with a photoinitiator, 0.15 mL of DOX solution, and 0.15 mL of RO-3306 solution were mixed evenly. Then, 0.15 mL of a 25% mass fraction of nano-iron oxide dispersion was added. The mixture was placed on a test tube shaker to mix evenly, obtaining the dispersed phase fluid required for the experiment. Two 10 mL disposable syringes were taken, and 10 mL of continuous phase fluid was drawn into each. Another 1 mL of dispersed phase fluid was drawn. The flow rates of the micro-injection pumps controlling the continuous phase fluid and the dispersed phase fluid were adjusted, and the two micro-injection pumps were started simultaneously. The dispersed phase fluid and the continuous phase fluid were injected into the flow focusing device from the syringe through the plastic capillary tube. When uniform microdroplets were formed at the outlet of the device, the plastic capillary tube at the outlet of the microchannel was placed in the oil phase of the collection beaker to obtain the uncured magnetic drug-loaded gelatin microspheres. After collection, the beaker was placed under a UV lamp for 20 min to solidify the hydrogel. The solidified gelatin microspheres were placed in a test tube, centrifuged and washed three times to remove the oil phase, and the dispersion of magnetic gelatin microrobots was obtained. The dispersion was covered with tin foil to block light and stored in a refrigerator at 4°C (Figure 1A).

### 2.9 Setup of magnetic field

The magnetic field generator is composed of a three-degree-of-freedom Helmholtz coil, a multifunction data acquisition unit (DAQ, NI-PCI-6259), and three single-channel output power amplifiers. By controlling the current and voltage of the Helmholtz coil from the driving signal amplified by voltage amplifiers, an external rotating uniform magnetic field can be generated in 3D space to manipulate the hydrogel microrobots. The Helmholtz coil is positioned on the observation platform of the microscope, enabling real-time observation of the swimming hydrogel microrobots.

The motion performance of the microrobots was investigated under an external magnetic field. A series of control experiments were conducted to elucidate the effect of different drive frequencies and the strength of the magnetic field on the velocity of the microrobots. Subsequently, the microrobots were navigated using a control strategy of a three-dimensional rotating magnetic field generated by the three-degree-of-freedom Helmholtz coil. In the x–z plane, the microrobot moved around the short axis (x-axis), applying a magnetic field given by B (t) = B0 [cos (ωt)ex + sin (ωt)] ez (where B0 is the magnitude, ω is the angular frequency of the magnetic field, and t is the time). Here, ex and ez represent the unit vector along the *x* and *z* axes, respectively (hereafter, ey represents that along the y axis). In the y–z plane, the microrobots moved around the long axis (y-axis), applying a magnetic field by B (t) = B0 [cos (ωt)ey + sin (ωt)] ez. By controlling the input of current, the direction of the rotating magnetic field can be altered to control the direction of the microrobot. Based on the dynamic control scheme above, a magnetically controlled microrobot could follow a predefined trajectory.

### 2.10 *In Vitro* simulation of drug inhibition loaded by magnetic-driven hydrogel microrobots

We introduce microrobots, loaded with Ro-3306 and doxorubicin, into a cell culture dish (35 mm in diameter) containing a complete culture medium. A directional magnetic field is then applied to the microrobots for 2 min, guiding them to the center of the dish. This process emulates the navigational capabilities of the microrobots and their precision drug delivery system. For cells cultivated under two-dimensional conditions, we employ PI staining to detect cellular responses 72 h post drug release. Specifically, after aspirating the culture medium and rinsing the cells with PBS, we incubate them in a solution of 4.5 μM PI in PBS at 37°C for 30 min. Following incubation, the cells are washed with PBS and immediately observed under a fluorescence microscope. Dead cells are identified by their red fluorescence (PI). Imaging is performed using a Leica TCS SP8 laser scanning confocal microscope (Leica, United States), and the captured images are processed using the Leica Application Suite X (LAS X; Leica, United States). For cells grown under three-dimensional conditions, we capture images of green fluorescence with the Leica TCS SP8 laser scanning confocal microscope (Leica, United States) and process them using the Leica Application Suite X (LAS X; Leica, United States). Additionally, we employ the CellTiter-Glo Luminescent Cell Viability Assay (as previously described) to assess cell viability under three-dimensional culture conditions (Figure 1B).

### 2.11 Statistical analysis

Details of statistical analyses of the various experiments are described in the relevant methods section. If not specified, statistical analysis was carried out using GraphPad Prism 8 software (GraphPad Software, United States). After confirming that values followed a normal distribution, two-tailed Student’s t test was applied to determine the significance of differences between two groups of independent samples. Pearson’s correlation analysis and Spearman’s correlation analysis were performed to determine the correlation between two group of variables.

## 3 Results

### 3.1 CDK1 inhibition inhibits osteosarcoma cell proliferation and promote chemotherapy sensitivity

**FIGURE 2:**
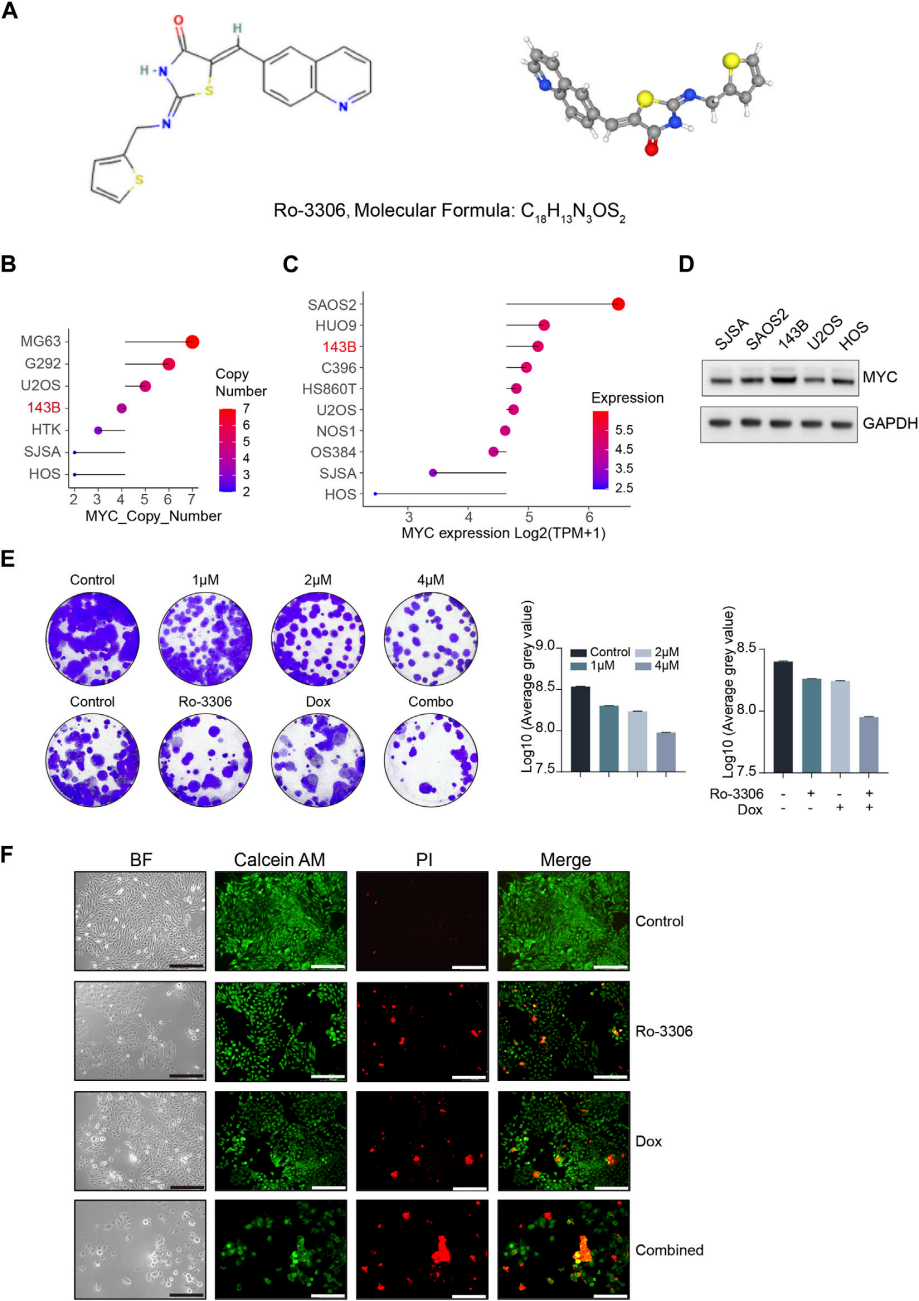
Ro-3306 effectively curtails the proliferation of osteosarcoma cells and enhances the therapeutic potency of doxorubicin under 2D cell culture. **(A)** Two-dimensional (2D) and three-dimensional (3D) chemical structure features of Ro-3306. 2D structure image of CID 135400873 (Ro-3306), PubChem Identifier: CID 135400873, URL: https://pubchem.ncbi.nlm.nih.gov/compound/Ro-3306. 3D structure image of CID 135400873 (Ro-3306), PubChem Identifier: CID 135400873, URL: https://pubchem.ncbi.nlm.nih.gov/compound/Ro-3306#section=3D-Conformer. **(B, C)** Absolute copy number of MYC and mRNA level of MYC [Log2 (TPM+1)] in common osteosarcoma cell lines in the CCLE database (https://sites.broadinstitute.org/ccle/) **(D)** The protein expression level of MYC in common osteosarcoma cells. **(E)** Colony formation assay to detect the proliferative ability of osteosarcoma cells after treatment with Ro-3306 (0, 1μM, 2μM, and 4 μM) and combined treatment with Ro-3306 and doxorubicin. **(F)** Osteosarcoma cell survival and regulation after drug treatment was detected by Calcein/AM-PI. The magnification is 100 times.

In normal cells, MYC carefully regulates transcription and DNA replication, while also facilitating DNA damage repair. However, when MYC is overexpressed, it triggers a cascade of cellular stress responses, leading to the transformation of normal cells into cancerous ones, thereby driving cancer progression and potentially affecting the efficacy of cancer treatments. Studies suggest that in tumors with Myc overexpression, the tumor cells become dependent on Myc. Therefore, targeting downstream targets of Myc could be more effective in treating cancer patients with MYC gene amplification. Tumor cells with MYC overexpression show dysregulation of the cell cycle, which could make their proliferation especially sensitive to the inhibition of cyclin-dependent kinase (CDK), a key regulator of cell cycle progression. Research has shown that the inhibition of CDK1 can induce MYC-dependent apoptosis. Furthermore, treating MYC-dependent mouse lymphoma and hepatoblastoma with CDK1 inhibitors has been shown to reduce tumor growth and prolong survival time (Goga et al., 2007). MYC-driven osteosarcoma is characterized by genomic amplification and high expression of the MYC gene. This subtype of osteosarcoma has the worst prognosis, with a 5-year survival rate of less than 40%. Improving the prognosis of patients with this subtype or overcoming the overall prognosis “bottleneck” of osteosarcoma is a critical step (Jiang et al., 2022). CDK1 may be a potential therapeutic target in this context. However, given the pivotal role of its synthetic lethal gene CDK1 in the regulation of the normal cell cycle and the lack of a specific target in osteosarcoma cells, the use of magnetically driven non-targeted robots could theoretically reduce the strong toxic effects on surrounding normal tissues and effectively increase the drug concentration in osteosarcoma tissues (Wang et al., 2023). The chemical structure and three-dimensional characteristics of Ro-3306 (Kim et al., 2023) are depicted in Figure 2A. We used the CCLE database to examine the changes in the copy number and mRNA expression of the MYC gene in osteosarcoma cell lines (Figures 2B,C), and selected SJSA, SAOS2, 143B, U2OS, and HOS - five types of human osteosarcoma cell lines - to detect MYC protein expression levels (Figure 2D). By integrating the changes in MYC at the gene, transcription, and protein levels, we chose the 143B human osteosarcoma cell line for subsequent MYC-dependent osteosarcoma cell model studies. Next, to illustrate the therapeutic potential of Ro-3306, we assessed the cytotoxic effect of Ro-3306 on osteosarcoma 143B cells under *in vitro* two-dimensional cell culture conditions. First, we determined the suppressive impact of Ro-3306 on the proliferative capacity of the osteosarcoma cell line 143B through a colony formation assay. The results showed that administering 1 μM, 2 μM, and 4 μM of Ro-3306 to the osteosarcoma cell line 143B could effectively inhibit its proliferative capacity, showing a concentration-dependent relationship. Considering the widespread occurrence of chemoresistance in osteosarcoma, we then evaluated whether Ro-3306 could enhance the chemotherapeutic effect of the osteosarcoma cell line. We used lower concentrations of doxorubicin (10 nM) and Ro-3306 (1 μM) for subsequent research. Accordingly, through the colony formation assay, a lower concentration of Ro-3306 successfully sensitized the therapeutic effect of low-dose doxorubicin, and the proliferative capacity of the osteosarcoma cell line 143B was significantly reduced (Figure 2E). Finally, Calcein/AM-PI double staining was used to evaluate the response of osteosarcoma 143B cell to Ro-3306 with or without the combination of doxorubicin. Compared with the control group, the cell survival rate of osteosarcoma cell treated with Ro-3306 or doxorubicin was significantly reduced. Moreover, the combination of Ro-3306 and doxorubicin administration strategy showed enhanced tumor-killing effect, indicating that this co-dosing strategy exerts a significant cytotoxic effect on osteosarcoma cell (Figure 2F). These findings suggest that Ro-3306 has potent cytotoxic effects on osteosarcoma cells and inhibits cell proliferation, and has the effect of sensitizing chemotherapy for osteosarcoma under 2D cell culture condition.

**FIGURE 3:**
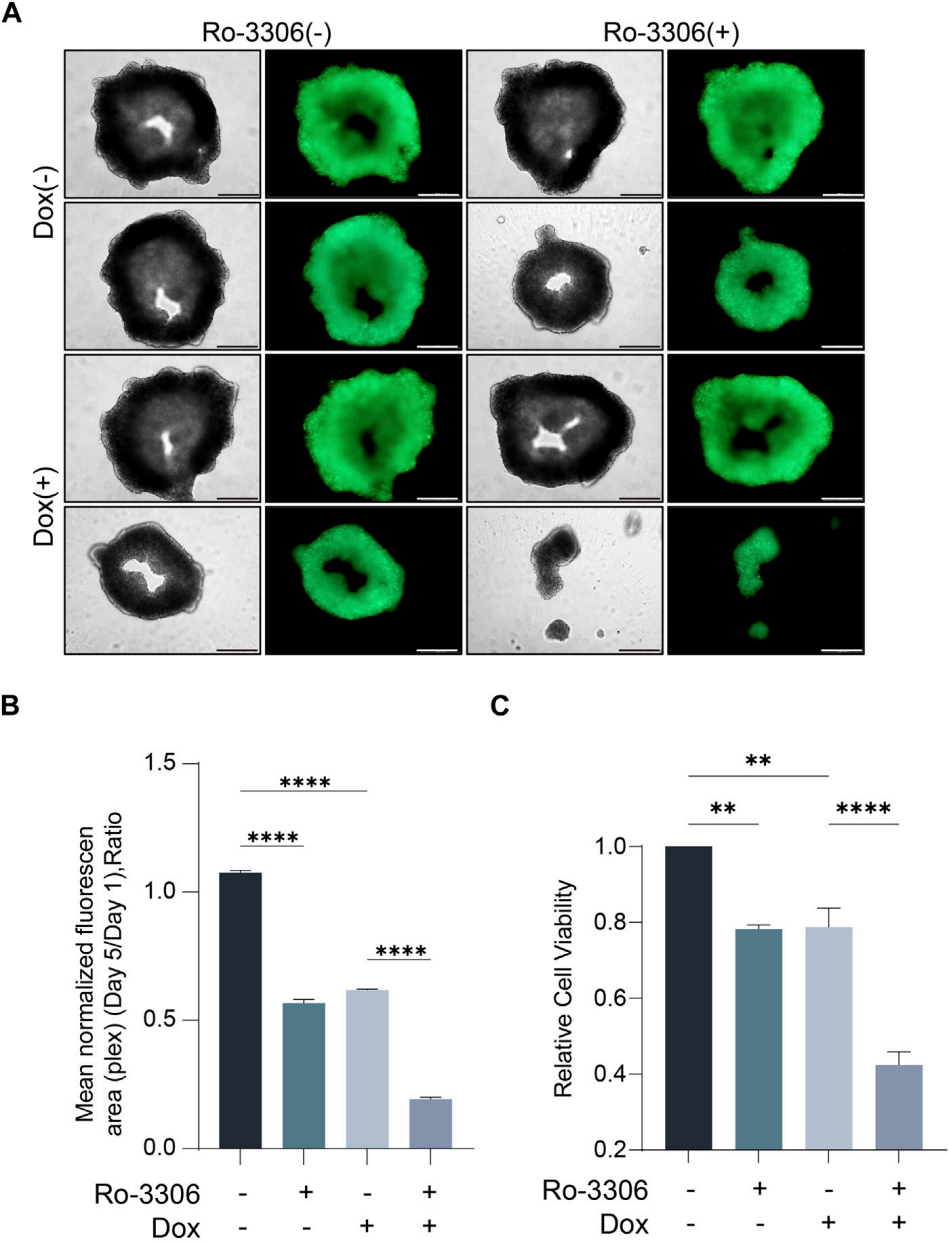
Ro-3306 effectively curtails the proliferation of osteosarcoma cells and enhances the therapeutic potency of doxorubicin under 3D cell culture. **(A)** Representative optical and fluorescence microscope images of osteosarcoma cells under three-dimensional spheroid culture conditions after combined treatment with Ro-3306 and doxorubicin. The magnification is 100 times. **(B)** Quantitative analysis of fluorescence intensity of osteosarcoma cells under three-dimensional spheroid culture conditions after combined treatment with Ro-3306 and doxorubicin. **(C)** CellTiter-Glo assay was applied to detect luminescence and cell viability of osteosarcoma cell lines treated with RO-3306+DOX combination strategy.

Given that cell morphology, intercellular interactions, and cell polarity often undergo alterations in the two-dimensional culture system, rendering it challenging to emulate the authentic growth of tumor cells *in vivo*, the predictability of preclinical research predicated on this is limited (Jubelin et al., 2022; Wang and Jeon, 2022). Three-dimensional cell culture, a novel cell culture methodology developed in recent years, can more accurately simulate the *in vivo* growth state of cells, and facilitate subsequent drug screening (Jensen and Teng, 2020; Gonzalez Diaz et al., 2022). Consequently, we selected three-dimensional cell spheroid culture for subsequent experiments. Employ-ing the same concentration of Ro-3306 and doxorubicin as in the two-dimensional cell colony formation experiment to treat the osteosarcoma cell line 143B under three-dimensional culture conditions, the results indicated that Ro-3306 effectively enhanced the chemotherapy sensitivity of the osteosarcoma cell line 143B under three-dimensional culture conditions, and the fluorescence intensity was significantly (Figures 3A,B) attenuated consistent with cell viability assays (Figure 3C).

### 3.2 Preparation of the magnetic driven hydrogel micro-robotic drug delivery system

**FIGURE 4:**
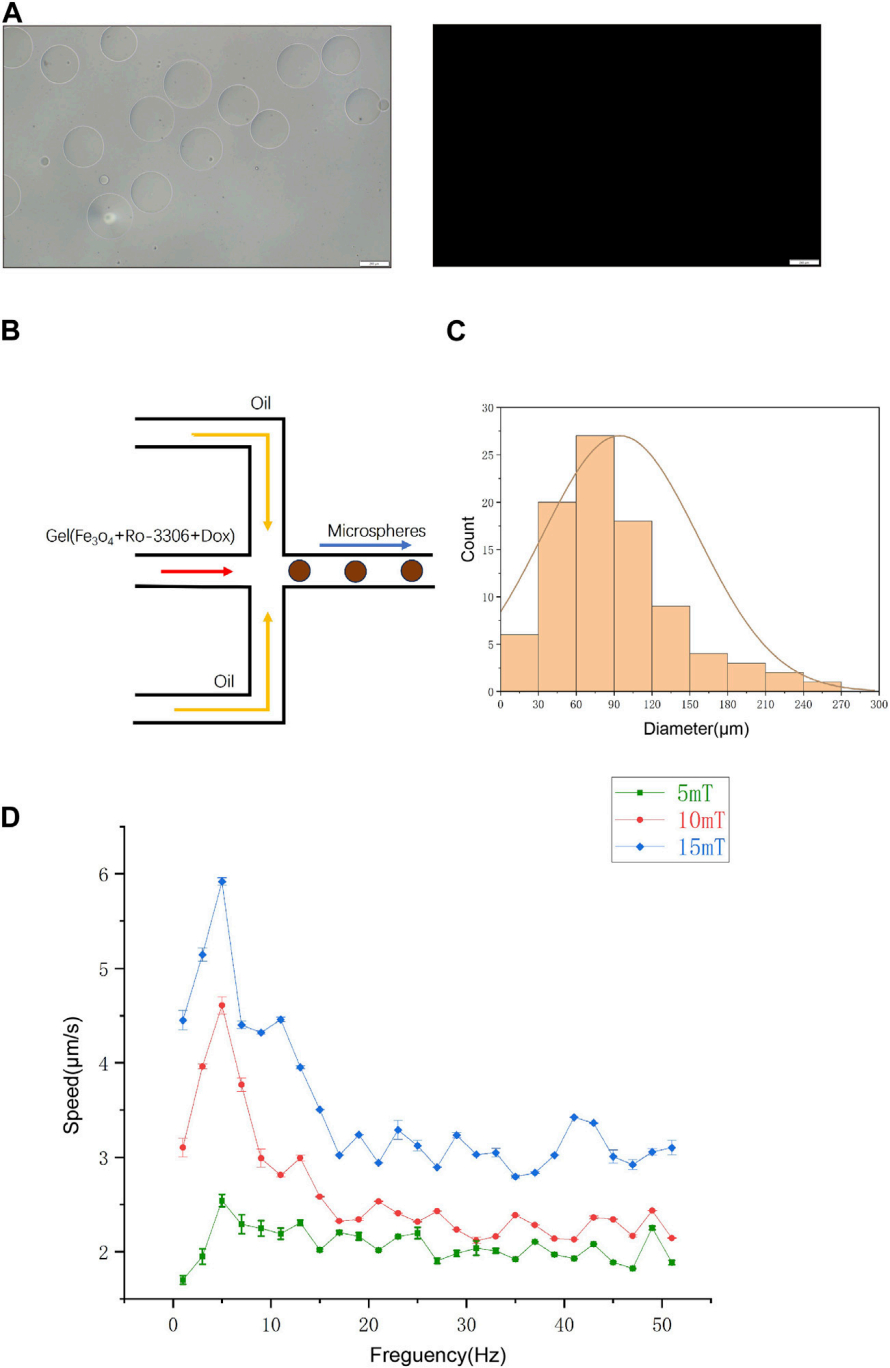
The orchestration of a targeted drug conveyance mechanism facilitated by the utilization of magnetic-driven hydrogel microrobots. **(A)** Optical and fluorescence microscopy images of Magnetic-driven hydrogel micro-robots without drug loading. **(B)** Process diagram of a microfluidic system constructing a drug-loaded magnetic-driven hydrogel microrobot. The dispersed phase fluid and the continuous phase fluid are separately injected into the flow focusing device through a plastic capillary tube from the syringe, collecting the uncured magnetic drug-loaded gelatin microspheres. After being irradiated by an ultraviolet lamp to solidify the gelatin, the oil phase is removed to obtain the dispersion of the magnetic gelatin microrobots, which is used for subsequent experiments. **(C)** Size distribution chart of Magnetic-driven hydrogel microrobot particles. **(D)** Motion rate characteristics of Magnetic-driven hydrogel micro-robots at frequencies from 2 to 52 Hz under different magnetic field densities.

Doxorubicin, a frontline chemotherapeutic agent for osteosarcoma, is widely used in clinical treatments, mainly administered via intravenous injection. However, doxorubicin, by integrating into DNA and inhibiting topoisomerase II, impedes DNA replication, thereby damaging normal tissue cells and causing a cascade of toxic side effects, including bone marrow suppression, cardiotoxicity, gastrointestinal disturbances, and neurotoxicity, among others. Drug delivery systems may effectively mitigate these toxic side effects. Among these, micro/nano robots, which can be controlled and regulated, are considered to have revolutionary innovative potential in precision-targeted drug delivery systems, due to their ability to propel and navigate within diverse liquid media. The use of magnetic-driven micro-robots in drug delivery is a growing research field. The primary objective is to precisely control the locomotion of micro-robots to deliver drugs with pinpoint accuracy to specific sites within the body. The applications in drug delivery, surgical procedures, diagnostics, and proactive tumor site targeting are emerging. Based on the chemosensitizing attributes of Ro-3306 in osteosarcoma cell lines, we describe an innovative magnetic-driven hydrogel micro-robot loaded with Ro-3306 and doxorubicin. The aim is to retain the drug, enabling it to reach the target location and effect its release through a precise drug delivery system under the influence of an external magnetic field, thereby enhancing the local therapeutic effect. In this process, the magnetic-driven hydrogel micro-robot exhibits remarkable magnetic responsiveness and a unique structure to ensure drug release performance. Initially, we fabricated the unloaded hydrogel micro-robot (without Fe_3_O_4_) (Figure 4A). After observing its morphological characteristics, we started drug loading. Figure 4B illustrates the specific process used to synthesize the magnetic-driven hydrogel micro-robot loaded with Ro-3306 and doxorubicin in this study. Magnetic-driven hydrogel micro-robots are fabricated using a microfluidic chip based on the principle of flow focusing. Within the confluence device, the dispersed phase fluid and the continuous phase fluid pass through a narrow region under pressure. At the microdroplet generation site, three fluid streams are present, with the continuous phase fluid symmetrically distributed on both sides. The central dispersed phase fluid is focused and sheared by the continuous phase fluid on both sides, resulting in the formation of microdroplets. Subsequently, the microdroplets are solidified under ultraviolet light exposure and coagulate on the surface, thus transforming into a magnetic-driven hydrogel micro-robot. During this process, the continuous phase consists of vegetable oil, and the dispersed phase contains Ro-3306, doxorubicin, and Fe_3_O_4_ particles. Figure 4C shows the statistical analysis of the size distribution of the fabricated micro-robots, and the findings indicate that the particle size distribution follows a normal distribution. According to the particle size distribution chart, the size range of the hydrogel microrobots can be determined to be 20–270 μm, mainly concentrated between 30 and 120 μm. In addition, regarding drug loading, the concentration of RO-3306 drug loaded inside the hydrogel microrobots is 2μM, while the DOX drug concentration is 20 nM. After the assembly of the magnetic-driven hydrogel micro-robots is completed, we examined the locomotion of the magnetic-driven hydrogel micro-robot under different magnetic field strengths. By increasing the strength and frequency of the magnetic field, the velocity of the micro-robot rises more quickly, reaching a peak speed of 6 μm/s (Figure 4D). Under normal circumstances, an increase in magnetic field frequency will lead to an increase in the motion speed of the hydrogel microspheres. This is because the magnetic moment of the microspheres will follow the changes in the magnetic field, thereby generating a propulsive force, causing the microspheres to accelerate. However, when the magnetic field frequency exceeds a certain value, the motion of the microspheres will lose synchronization, leading to a decrease in motion speed. This phenomenon is known as the out-of-step phenomenon, which is due to the inability of the microsphere’s magnetic moment to follow the changes in the magnetic field in time under a high-frequency magnetic field, causing the microsphere to fail to generate effective propulsion with the changes in the magnetic field, thereby leading to a decrease in motion speed. Taken together, our research results demonstrate that the magnetic-driven hydrogel micro-robot constructed in this study exhibits controllable motion patterns. At the same time, Ro-3306 enhances the chemosensitivity of osteosarcoma under both two-dimensional and three-dimensional cell culture conditions. Future research will focus on establishing a precision-targeted drug delivery system for Ro-3306 and doxorubicin, based on the platform of the magnetic-driven hydrogel micro-robot.

**FIGURE 5:**
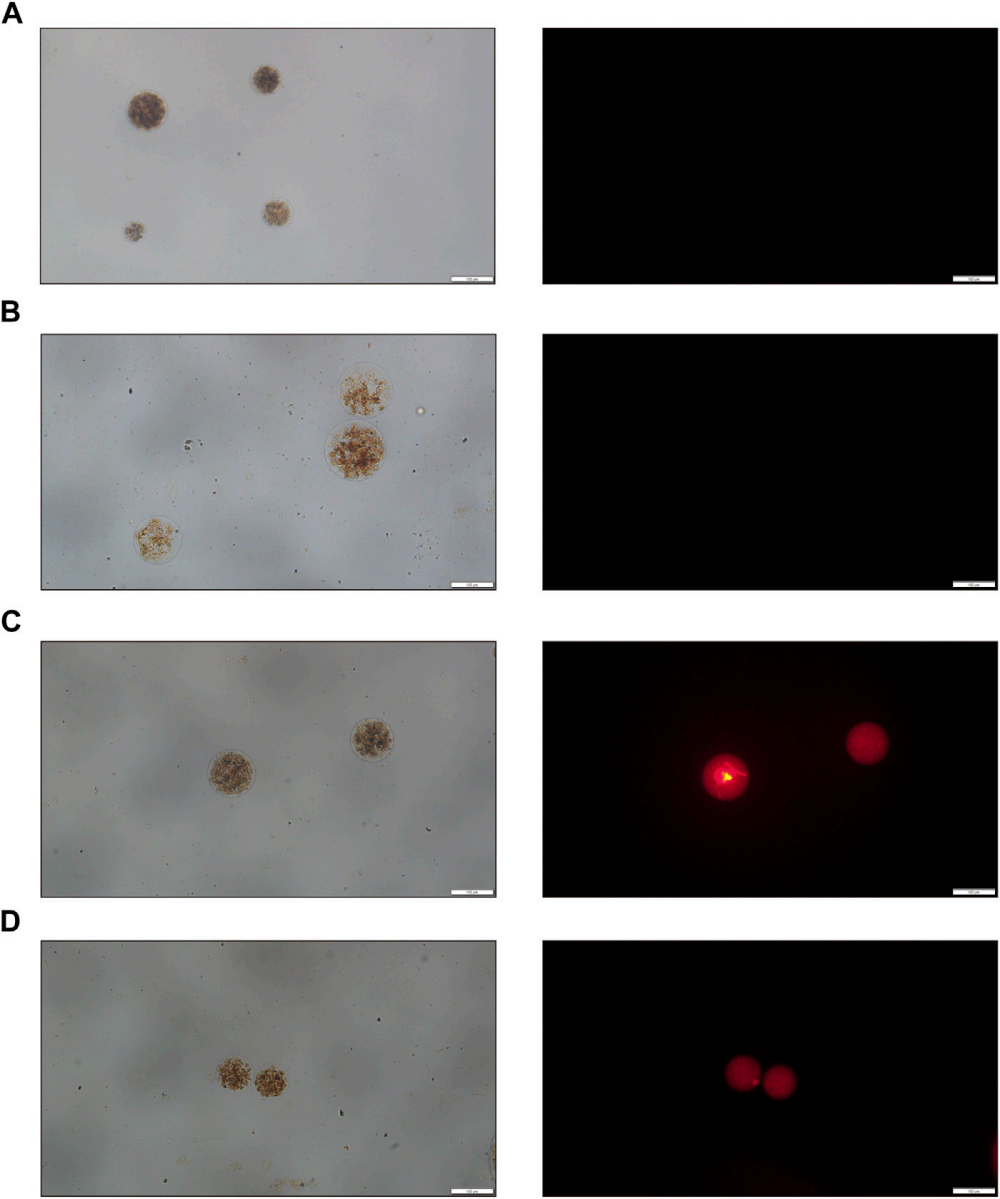
Optical and fluorescence microscopy images of drug-loaded magnetic-driven hydrogel micro-robots. **(A)** Optical and fluorescence microscopy images of Magnetic-driven hydrogel micro-robots without drug loading. **(B)** Optical and fluorescence microscopy images of Ro-3306-loaded magnetic-driven hydrogel micro-robots. **(C)** Optical and fluorescence microscopy images of Dox-loaded magnetic-driven hydrogel micro-robots. **(D)** Optical and fluorescence microscopy images of magnetic-driven hydrogel micro-robots loaded with Ro-3306 and Dox.

Initially, we use the intrinsic fluorescence properties of doxorubicin and a fluorescence microscope to examine the characteristics of drug loading. Under bright field visualization, both the unloaded and Ro-3306-loaded magnetic-driven hydrogel micro-robots appear as a brown color and show no fluorescence (Figures 5A, B). The doxorubicin-loaded magnetic-driven hydrogel micro-robot exhibits characteristic red fluorescence under a fluorescence microscope (Figure 5C). At the same time, the magnetic-driven hydrogel micro-robot loaded with both Ro-3306 and doxorubicin shows no significant difference from other components under bright field conditions, and the red fluorescence intensity within the micro-robot structure, as observed under a fluorescence microscope, is reduced (Figure 5D). These findings indicate that both Ro-3306 and doxorubicin have been successfully incorporated into the micro-robots, either individually or in combination.

### 3.3 *In vitro* stimulation of magnetic-driven hydrogel micro-robots to enhance osteosarcoma chemo-therapy with synthetic lethality strategy

**FIGURE 6:**
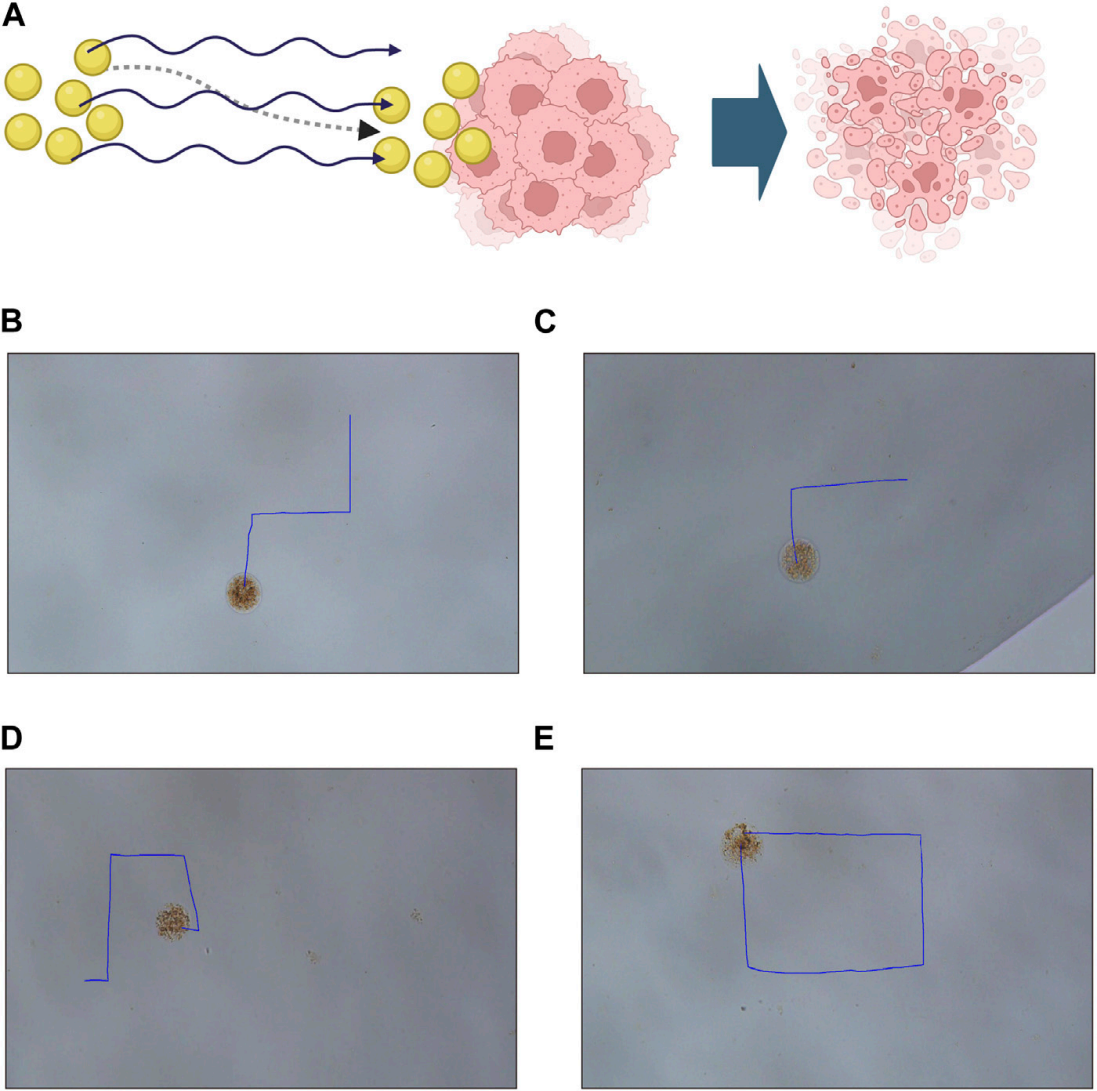
Motion characteristics and controllable flexible motion performance of Magnetic-driven hydrogel micro-robots. **(A)** Schematic illustration of tumor destruction mediated by a drug-loaded delivery system facilitated by magnetic-driven hydrogel microrobots. Created with BioRender.com **(B)** Controllable motion modes of Magnetic-driven hydrogel micro-robots without drug loading. **(C)** Controllable motion modes of Ro-3306-loaded Magnetic-driven hydrogel micro-robots. **(D)** Controllable motion modes of Dox-loaded Magnetic-driven hydrogel micro-robots. **(E)** Controllable motion modes of Magnetic-driven hydrogel micro-robots loaded with Ro-3306 and Dox.

An effective drug targeting delivery system depends on the controllable motion of the drug-loaded system and its efficient drug release capabilities. Therefore, we first examine the movement pattern of our constructed drug-loaded magnetic-driven hydrogel micro-robots, enabled by the application of an externally controllable magnetic field (Figure 6A). By adjusting the orientation of the magnetic field, the fine-tuned movement of the magnetic-driven hydrogel micro-robot can be achieved. As shown in Figures 6B–E (Supplementary Videos S1-S4), by changing the direction of the magnetic field, controlled regular motion trajectories of the magnetic-driven hydrogel micro-robot loaded with drug-free, Ro-3306, Doxorubicin, and both drugs were achieved.

**FIGURE 7:**
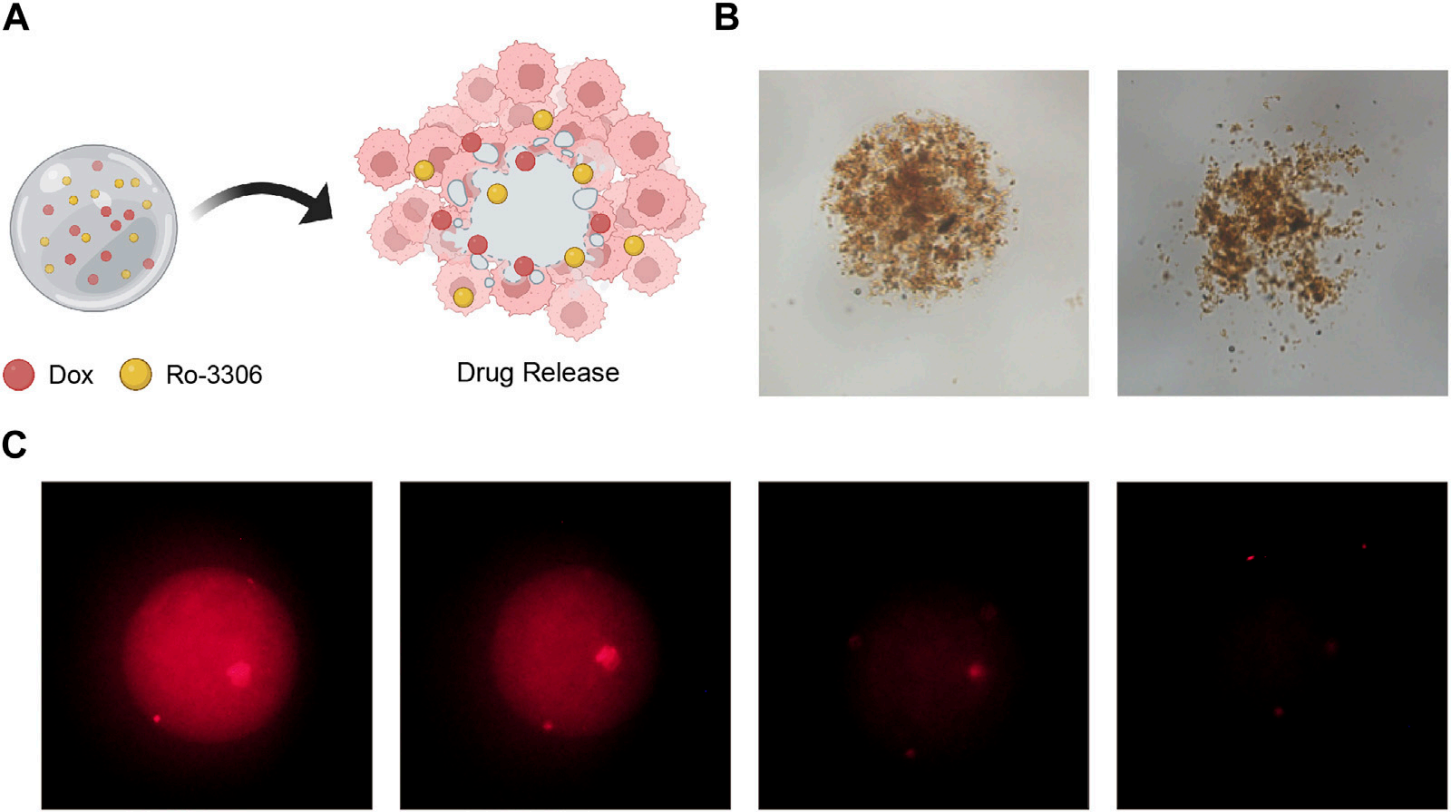
Drug release performance of drug-loaded Magnetic-driven hydrogel micro-robots. **(A)** Schematic illustration of drug release from drug-loaded Magnetic-driven hydrogel micro-robots. Created with BioRender.com. **(B)** Optical microscopy images of drug release from drug-loaded Magnetic-driven hydrogel micro-robots. **(C)** Fluorescence microscopy images of drug release from drug-loaded Magnetic-driven hydrogel micro-robots.

Subsequently, to illustrate the efficacy of our magnetic-driven hydrogel micro-robot in the field of drug delivery, we monitored the changes in drug release over various time intervals (Figure 7A). Using both bright field and fluorescence microscopy, we observed that the drug-loaded magnetic-driven hydrogel micro-robot exhibited consistent drug release capabilities as time elapsed (Figures 7B,C; Supplementary Video S5). These results suggest that the synthesized magnetic-driven hydrogel micro-robot loaded with Ro-3306 and Doxorubicin in this study can perform fine movements under the control of an external magnetic field, enhancing the transport capacity of the drug and providing a solid basis for precise drug delivery methods.

**FIGURE 8:**
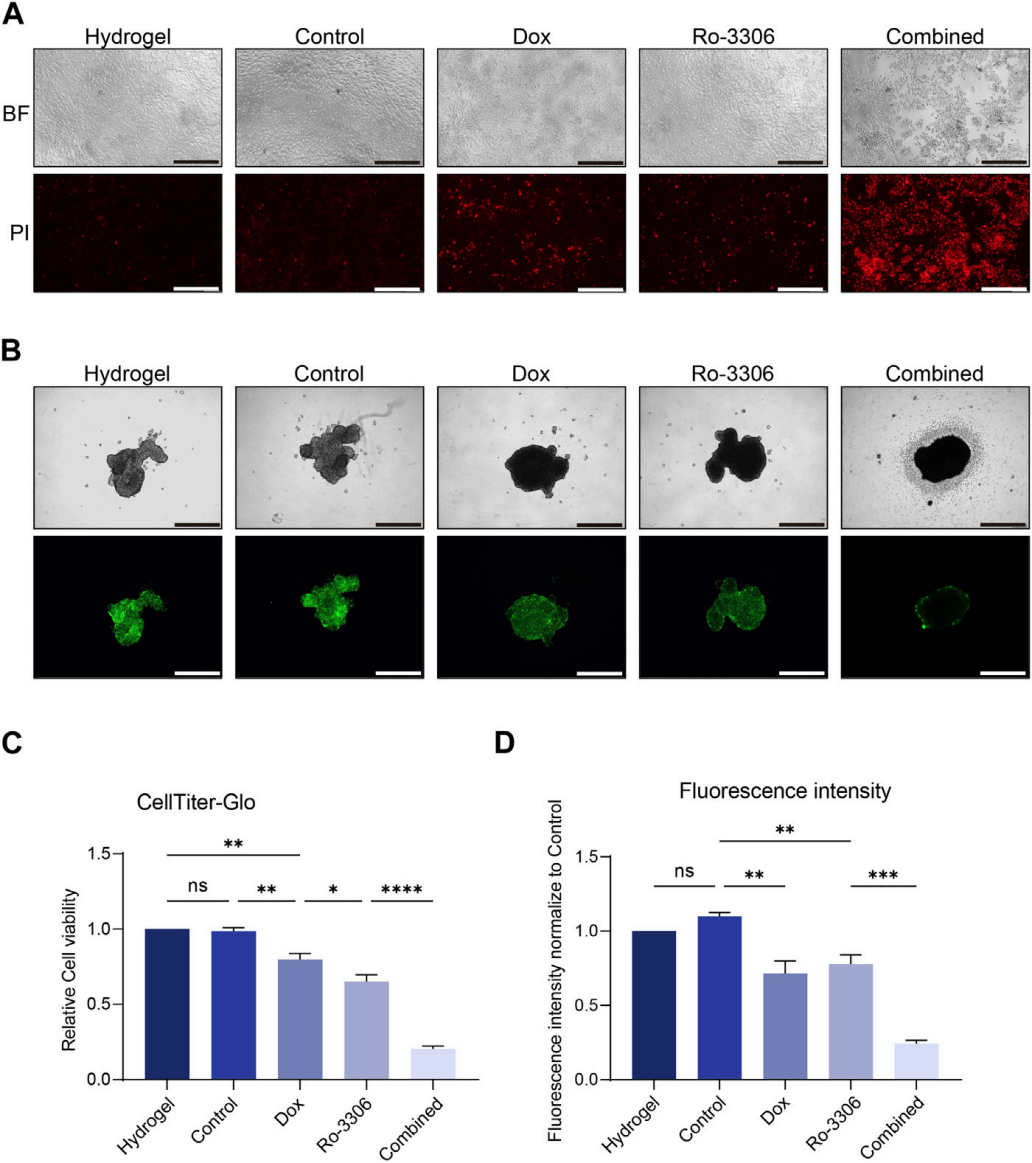
Drug-loaded Magnetic-driven hydrogel micro-robots exhibit potent cytotoxicity against osteosarcoma cells (under both two-dimensional and three-dimensional cell culture conditions) through the release of drugs, thereby demonstrating a pronounced synergistic effect. **(A)** Representative optical and fluorescence microscope images of osteosarcoma cells under two-dimensional culture conditions following a combined treatment regimen with Ro-3306 and doxorubicin. PI staining is employed to discern the cytotoxic effects exerted by both unloaded Magnetic-driven hydrogel micro-robots and drug-loaded Magnetic-driven hydrogel micro-robots on osteosarcoma cells. The magnification is 100 times. **(B)** Representative optical and fluorescence microscope images of osteosarcoma cells under three-dimensional culture conditions after combined treatment with Ro-3306 and doxorubicin. The magnification is 100 times. **(C)** Quantitative analysis of fluorescence intensity of osteosarcoma cells under three-dimensional spheroid culture conditions after combined treatment with Ro-3306 and doxorubicin loaded by Magnetic-driven hydrogel micro-robots. **(D)** CellTiter-Glo assay was applied to detect luminescence and cell viability of osteosarcoma cell lines treated with RO-3306+DOX combination strategy loaded by Magnetic-driven hydrogel micro-.robots.

Finally, we simulated the tumoricidal effect of the drug-loaded magnetic-driven hydrogel micro-robots’ delivery system *in vitro* (Figure 1B). By applying a directional magnetic field to the drug-loaded magnetic-driven hydrogel micro-robots for 2 min, we moved them to the center of the culture dish under both two-dimensional and three-dimensional cell culture conditions. This process mimics the navigational capabilities of the micro-robots and their precision drug delivery system. For cells cultured under two-dimensional conditions, we used PI staining to identify dead cells 72 h after drug release (Figure 8A). For cells under three-dimensional culture conditions, we measured the expression of green fluorescence (Figures 8B,C) and ATP activity (CellTiter-Glo Luminescent Cell Viability Assay) to evaluate the drug’s cytotoxic effect (Figure 8D). Our *in vitro* experiments show that these magnetically driven micro-robots can effectively deliver drugs and inhibit tumor growth.

## 4 Discussion

This study is focused on investigating the therapeutic potential of CDK1 inhibitors for the treatment of MYC-dependent osteosarcoma. We have designed and fabricated drug-loaded, magnetic-driven hydrogel micro-robots, and applied them to MYC-dependent osteosarcoma cells, thereby implementing a synthetic lethal strategy that targets CDK1. Through the use of both two-dimensional and three-dimensional cell culture models, we have demonstrated that our drug delivery system, which is based on magnetic-driven hydrogel micro-robots, can effectively eradicate tumor cells and enhance the chemosensitivity of MYC-dependent osteosarcoma cells. Our research indicates that drug-loaded, magnetic-driven hydrogel micro-robots can mitigate the toxic side effects of CDK1 inhibitors on normal tissue cells, and can also reduce the dosage of doxorubicin through a combined medication strategy.

Drug Delivery Systems (DDS) are technologies designed to deliver drugs effectively to targeted sites. They can enhance the efficacy of drugs, minimize the side effects, prolong the duration of drug action, and improve the bioavailability of drugs (Yang et al., 2022). Magnetic-driven hydrogel micro-robots (MMHR) are a type of micro-robot that is based on hydrogels. The propulsion of hydrogel microspheres primarily relies on the rotating magnetic field, which can generate a strong propulsive force. In low Reynolds number fluids, the resistance experienced by the microspheres is relatively small, and the interference from Brownian motion can be ignored. They possess several unique advantages. Firstly, MMHR can utilize an external magnetic field to achieve precise motion control and navigation, thereby overcoming the fluid dynamic resistance and interference from Brownian motion in low Reynolds number fluids. Secondly, MMHR can leverage the high water absorption and porosity of hydrogels to achieve efficient drug loading and responsive drug release, thus avoiding premature drug leakage and non-specific effects. Thirdly, MMHR can take advantage of the biocompatibility and biodegradability of hydrogels to reduce toxicity and side effects on the organism, thereby enhancing safety and bioavailability. Lastly, MMHR can utilize the plasticity and programmability of hydrogels to design various shapes and functions, such as cylindrical, hexahedral, spiral, spherical, capsule shapes, etc., to adapt to different drug delivery routes and target sites (Vargason et al., 2021; Jiang et al., 2022).

Osteosarcoma is a malignant tumor that manifests in the bones, predominantly affecting children and adolescents. The primary treatment strategies for osteosarcoma hinge on surgical resection and chemotherapy. However, due to the pronounced heterogeneity and drug resistance of osteosarcoma, the efficacy of chemotherapy is suboptimal, leading to a high rate of recurrence and metastasis, and consequently, a poor prognosis. Therefore, the identification of novel therapeutic targets and strategies is crucial for enhancing the survival rate of osteosarcoma patients (Shoaib et al., 2022). The Synthetic Lethal Strategy (SLS) is a therapeutic approach that exploits the unique genetic defects in tumor cells to selectively induce their death, without impacting the survival of normal cells. The success of SLS lies in its ability to accurately deliver drugs to the tumor site, thereby minimizing damage to normal tissues. In recent years, SLS has been successfully employed in the treatment of various cancers. For instance, MTAP-deficient tumors have shown improved treatment outcomes in various cancers such as pancreatic cancer and glioblastoma by utilizing PRMT5 as a synthetic lethal target (Huang et al., 2020; Alhalabi et al., 2022). Osteosarcoma is characterized by various genetic defects, among which the amplification of the oncogene MYC is considered a significant factor contributing to a poor prognosis. This genetic defect renders osteosarcoma cells dependent on certain genes or pathways, thereby offering potential targets for SLS. MYC is a transcription factor encoded by the oncogene *myc*, which regulates processes such as cell growth, proliferation, and metabolism. The amplification or overexpression of MYC in various tumors is associated with tumor invasiveness, metastasis, and prognosis. However, MYC itself is a challenging protein to target, necessitating the identification of synthetic lethal genes that cooperate with MYC. Research has revealed that tumor cells with high MYC expression exhibit enhanced dependence on CDK1. The inhibition of CDK1 can induce apoptosis in cells with high MYC expression, without affecting the survival of cells with low MYC expression (Sayles et al., 2019).

We have successfully engineered a magnetic-driven hydrogel micro-robot loaded with Ro-3306, establishing an efficient drug delivery system. We have confirmed the sensitivity of MYC-dependent osteosarcoma cells to CDK1 inhibition *in vitro* and have successfully enhanced the chemotherapeutic responsiveness of these cells. Consequently, our magnetic-driven hydrogel micro-robot drug delivery system shows promise for the treatment of MYC-dependent osteosarcoma. However, in clinical settings, patients with osteosarcoma often bear a substantial tumor burden. Whether the micro-robot can overcome blood flow resistance to reach the tumor site, and whether it can penetrate deeper parts of the primary tumor, are questions that warrant further investigation. To address this issue, three strategies can be employed. Firstly, we can modify the surface with specific ligands or antibodies that have the ability to bind to particular receptors present on the surface of tumor cells. Secondly, we can utilize high-precision navigation systems, which may include magnetic, chemical gradient, and optical navigation. These systems can be used either individually or in combination to achieve our goal. Lastly, we can design a system capable of triggering drug release under specific conditions, such as certain pH levels, temperatures, or in the presence of specific biomolecules. Further investigation is required to ascertain the biocompatibility and safety of magnetic-driven hydrogel micro-robots. Future studies will employ *in vitro* experiments to expose these micro-robots to a variety of cell types, with the aim of determining whether they induce any toxic or immune responses. Additionally, their compatibility within a blood environment will be assessed by monitoring for any signs of blood clotting. The distribution and movement of these micro-robots within the body will be tracked using *in vivo* experiments in conjunction with imaging techniques such as MRI or ultrasound. Our ongoing research aims to verify whether this synthetic lethal drug-loaded magnetic-driven hydrogel micro-robot can inhibit the *in vivo* growth of osteosarcoma under the guidance of genomic features and the specific mechanism of CDK1 inhibition in sensitizing osteosarcoma chemotherapy.

Efficient Drug Delivery Systems (DDS) are employed in cancer treatment with the objective of achieving targeted therapy, i.e., delivering drugs accurately to the tumor site, thereby minimizing damage to normal tissues. This advantage can mitigate the toxic side effects that the Synthetic Lethal Strategy (SLS) may inflict on normal tissue cells. DDS and SLS hold significant research value in cancer treatment. They can offer new strategies and methods for treating various types of tumors, thereby improving the survival rate and quality of life of cancer patients. However, the application of DDS and SLS in cancer treatment still faces several challenges. For instance, improving the stability, biocompatibility, drug load, drug release rate, and tumor targeting of DDS are issues that need to be addressed in the design and optimization of DDS. Additionally, efficiently and accurately identifying gene pairs with synthetic lethal relationships, and verifying their mechanisms of action and effects in tumor cells, are challenges that need to be addressed in the screening and verification of SLS. Future research can focus on developing novel types of DDS, such as intelligent delivery systems, multifunctional delivery systems, and combination delivery systems, to enhance the performance and functionality of DDS. Moreover, exploring new SLS, such as those based on metabolism, signaling pathways, and epigenetics, can broaden the scope and applicability of SLS. With advancements in osteosarcoma diagnosis and treatment technology and the in-depth study of the pathogenesis of osteosarcoma aided by genomics and other methods, the future of osteosarcoma treatment will undoubtedly feature refined and individualized treatment strategies. The refined selection of chemotherapy, surgery, gene, and immunotherapy will undoubtedly benefit osteosarcoma patients. DDS and SLS have demonstrated significant research value in the treatment of various tumors and are expected to provide new insights and methods for the precise and individualized treatment of osteosarcoma patients.

## Data Availability

The original contributions presented in the study are included in the article/Supplementary Material, further inquiries can be directed to the corresponding authors.
